# CD160-Associated CD8 T-Cell Functional Impairment Is Independent of PD-1 Expression

**DOI:** 10.1371/journal.ppat.1004380

**Published:** 2014-09-25

**Authors:** Selena Viganò, Riddhima Banga, Florence Bellanger, Céline Pellaton, Alex Farina, Denis Comte, Alexandre Harari, Matthieu Perreau

**Affiliations:** 1 Division of Immunology and Allergy, Department of Medicine, Lausanne University Hospital, Lausanne, Switzerland; 2 Swiss Vaccine Research Institute, Lausanne University Hospital, University of Lausanne, Lausanne, Switzerland; Emory University, United States of America

## Abstract

Expression of co-inhibitory molecules is generally associated with T-cell dysfunction in chronic viral infections such as HIV or HCV. However, their relative contribution in the T-cell impairment remains unclear. In the present study, we have evaluated the impact of the expression of co-inhibitory molecules such as 2B4, PD-1 and CD160 on the functions of CD8 T-cells specific to influenza, EBV and CMV. We show that CD8 T-cell populations expressing CD160, but not PD-1, had reduced proliferation capacity and perforin expression, thus indicating that the functional impairment in CD160^+^ CD8 T cells may be independent of PD-1 expression. The blockade of CD160/CD160-ligand interaction restored CD8 T-cell proliferation capacity, and the extent of restoration directly correlated with the *ex vivo* proportion of CD160^+^ CD8 T cells suggesting that CD160 negatively regulates TCR-mediated signaling. Furthermore, CD160 expression was not up-regulated upon T-cell activation or proliferation as compared to PD-1. Taken together, these results provide evidence that CD160-associated CD8 T-cell functional impairment is independent of PD-1 expression.

## Author Summary

T-cell immune response is regulated by a variety of molecules known as co-inhibitory receptors. The over expression of co-inhibitory receptors has been observed in several chronic viral infections such as HIV disease, and is found to be associated with severe T-cell dysfunction. Recent studies have demonstrated that the co-expression of several co-inhibitory receptors correlated with greater impairment of CD8 T cells. However, the relative contribution of individual co-inhibitory receptors to the regulation of T-cell functions remains unclear. In order to shed light on these issues, we have evaluated the influence of the expression of 3 major co-inhibitory receptors such as PD-1, 2B4 and CD160 on CD8 T-cell functions such as proliferation, cytokines production and expression of cytotoxic granules. We demonstrate that CD160-associated CD8 T-cell functional impairment is independent of PD-1 expression and that the blockade of CD160 signaling may partially restore CD8 T-cell functions.

## Introduction

Co-stimulatory and co-inhibitory molecules play a major role in the regulation of antigen-specific T-cell responses [1]. Following T-cell receptor (TCR) engagement, activation or inhibition of T-cell responses depends upon the balance between stimulatory and inhibitory signals, on the type of molecules engaged or ligands involved and the availability of signaling molecules [2]–[4].

Co-stimulatory/co-inhibitory molecules are commonly divided into 4 families: 1) the B7 family including CD28, Cytotoxic T-lymphocyte associated protein-4 (CTLA-4), Programmed Death receptor-1 (PD-1), Inducible T-cell Costimulator (ICOS) and B- and T-lymphocyte attenuator (BTLA), 2) TNF-α receptor family including CD27, 3) the CD2/SLAM family, including Signaling Lymphocyte Activation Molecule (SLAM), 2B4 and CD48 and 4) the immunoglobulin (Ig) family including T-cell Immunoglobulin mucin-3 (TIM-3), lymphocyte Activation Gene-3 (LAG-3) and CD160 [5]–[10]. Each co-inhibitory/stimulatory molecule interacts with one or several receptors expressed by one or various cell types (reviewed in [2]).

During the past decade, many studies performed in mice and humans have underscored the role of co-inhibitory molecules in the functional impairment (also called “exhaustion”) of antigen-specific T cells during chronic viral infections such as human immunodeficiency virus-1 (HIV-1) or hepatitis C virus (HCV) [11]–[14]. In these virus chronic infections, the early functional impairment of T cells was marked by the loss of proliferation capacity likely resulting from reduced capacity to produce IL-2 and a deficient killing capacity of CD8 T cells. The ability to produce TNF-α was generally observed at an intermediate state of T-cell exhaustion while the loss of IFN-γ occurred in the advanced stage of T-cell exhaustion [15], [16].

Recent studies have demonstrated that HIV-specific CD8 T cells co-expressing several co-inhibitory molecules such as PD-1, CD160 and 2B4 were significantly more functionally impaired than CD8 T cells expressing only one co-inhibitory molecule [17]–[19]. However, the relative contribution of each co-inhibitory molecule has not yet been fully delineated.

In the present study, we evaluated the impact of the expression of co-inhibitory molecules such as 2B4, PD-1 and CD160 on CD8 T-cells specific to influenza (Flu), Epstein Barr virus (EBV) and cytomegalovirus (CMV). We demonstrated that CD160^+^ CD8 T cells had reduced proliferation capacity, IL-2 production and perforin expression regardless of PD-1 expression thus providing evidence that CD160-associated T-cell impairment is independent of PD-1.

## Results

### EBV and CMV-specific CD8 T cells express significantly higher levels of CD160 than Flu-specific CD8 T cells

**Figure 1:**
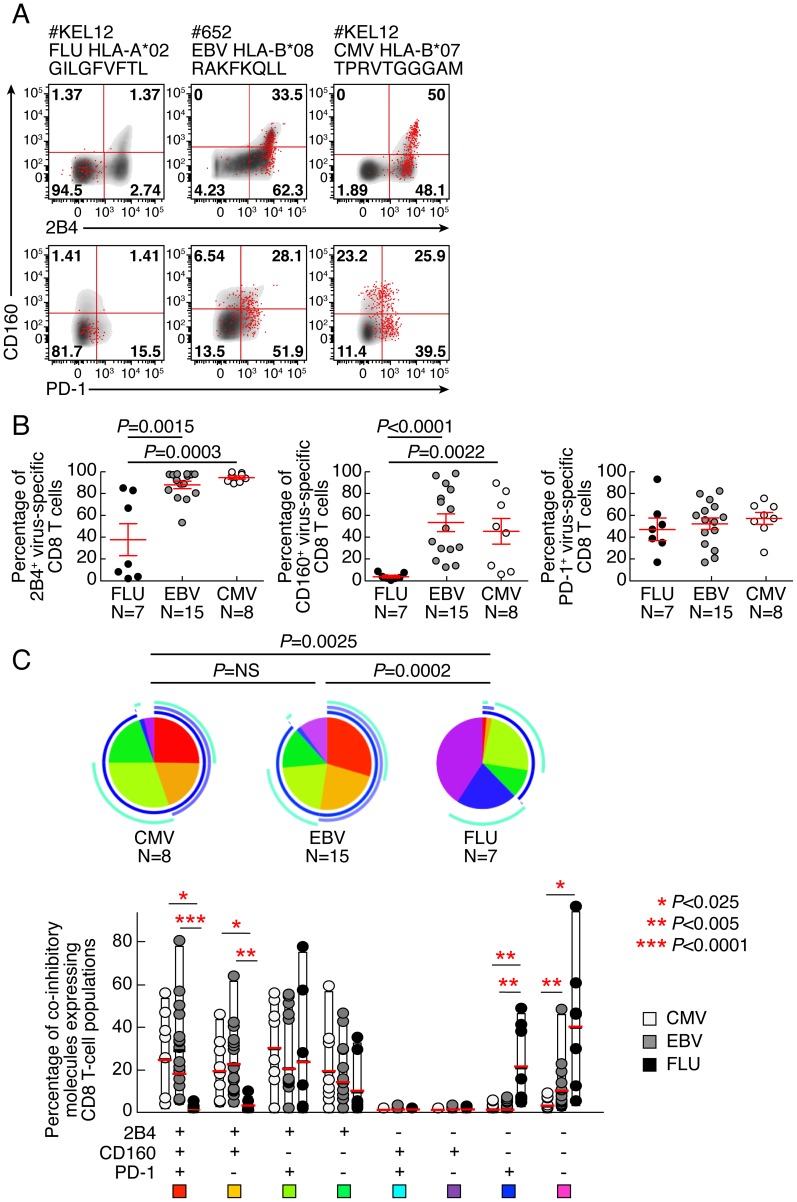
EBV and CMV-specific CD8 T cells express significantly more CD160 than Flu-specific CD8 T cells. Flow cytometric profiles of Flu, EBV and CMV-specific CD8 T cells expressing PD-1, CD160 and 2B4 *ex vivo*. The expression profiles of CD8 T cells were performed in 22 individuals using polychromatic flow cytometry. **(A)** 2B4, CD160 and PD-1 expression profiles of Flu, EBV and CMV-specific CD8 T cells detected by multimer staining (red) as compared to total CD8 T cells (black/grey) of three representative individuals (#KEL12 FLU HLA-A*02 GILGFVFTL, #652 EBV HLA-B*08 RAKFKQLL and #KEL12 CMV HLA-B*07 TPRVTGGGAM, respectively). **(B)** Frequencies of Flu, EBV and CMV-specific CD8 T cells expressing 2B4, CD160, PD-1. Red bars correspond to mean ± SEM. Statistical significance (*P* values) in panel **B** were obtained using One-way ANOVA (Kruskal-Wallis test) followed by a unpaired Student's t-test. **(C)** Expression profiles of 2B4, CD160, PD-1 of CMV, EBV and Flu-specific CD8 T cells. All the possible combinations of 2B4, CD160 and PD-1 expression are shown on the x axis and frequencies of 2B4, CD160 and PD-1 expression on CD8 and T-cell populations are shown on the y axis. Combinations of expression are grouped and color-coded on the basis of the number of molecules expressed. The pie chart summarizes the data, and each slice corresponds to the fraction of T cells expressing a given combination of molecules within the CD8 T-cell populations. Bars correspond to the fractions of distinct T-cell populations within the total T cells. Stars indicate statistical significance (*:*P*<0.025; **:*P*<0,005; ***:*P*<0.0001) and were calculated using the SPICE software.

The expression of PD-1, 2B4, and CD160 co-inhibitory molecules was assessed by multiparametric flow cytometry in CMV-, EBV- and Flu-specific CD8 T cells from 22 healthy individuals *ex vivo* using peptide-MHC class I multimer complexes (Table 1). The gating strategy defining the positivity of co-inhibitory molecule expression was set by fluorescence minus one (FMO) for all co-inhibitory molecules tested (Fig. S1). The use of 2B4, PD-1 and CD160 co-inhibitory molecules expression allowed the identification of 6 major CD8 T-cell populations *i.e.* 2B4^+^CD160^+^PD-1^+^, 2B4^+^CD160^+^PD-1^−^, 2B4^+^CD160^−^PD-1^+^, 2B4^+^CD160^−^PD-1^−^, 2B4^−^CD160^−^PD-1^+^ and 2B4^−^CD160^−^PD-1^−^ CD8 T-cell populations. The 2B4^−^CD160^+^PD-1^+^ and 2B4^−^CD160^+^PD-1^−^ CD8 T-cell populations are minor populations and represent less than 0.05% and 0.5% of virus-specific CD8 T cells, respectively, and were not included in the additional analyses shown below. Representative flow cytometry profiles and cumulative data (n = 30) showed that CMV and EBV-specific CD8 T cells expressed significantly higher levels of 2B4 (*P* = 0.0003 and *P* = 0.0015, respectively) and CD160 (*P*<0.0001 and *P* = 0.0022, respectively) as compared to Flu-specific CD8 T cells (Fig. 1A and B). Interestingly, no significant differences were observed in PD-1 expression between CMV, EBV and Flu-specific CD8 T cells in the cohort of subjects investigated in the present study (Fig. 1A and B). However, the proportion of 2B4^+^CD160^+^PD-1^+^ and 2B4^+^CD160^+^PD-1^−^ cells were significantly higher in CMV and EBV-specific CD8 T cells than in Flu-specific CD8 T cells (*P*<0.025) (Fig. 1C). Of note, mean fluorescent intensity (MFI) of CD2B4, CD160 and PD-1 was not significantly different between FLU, EBV and CMV-specific CD8 T cells (Fig. S2). Flu-specific CD8 T cells were mainly composed of 2B4^−^CD160^−^PD-1^−^, 2B4^−^CD160^−^PD-1^+^ and 2B4^+^CD160^−^PD-1^+^ CD8 T-cell populations (Fig. 1C). Interestingly, the co-inhibitory molecule expression profile of CMV and EBV-specific CD8 T cells was heterogeneous but not significantly different (*P*>0.05).

**Table 1:** Sequences of peptides used for CD8 T-cell stimulations and multimer staining.

Peptide sequences	HLA restriction	Virus
GILGFVFTL	A*02	Flu
RPPIFIRRL	A*02	EBV
RAKFKQLL	B*08	EBV
FLRGRAYGL	A*11	EBV
NLVPMVATV	A*02	CMV
TPRVTGGGAM	B*07	CMV

### CD160 but not PD-1 and/or 2B4 expression negatively correlates with CD8 T-cell proliferative capacity

**Figure 2:**
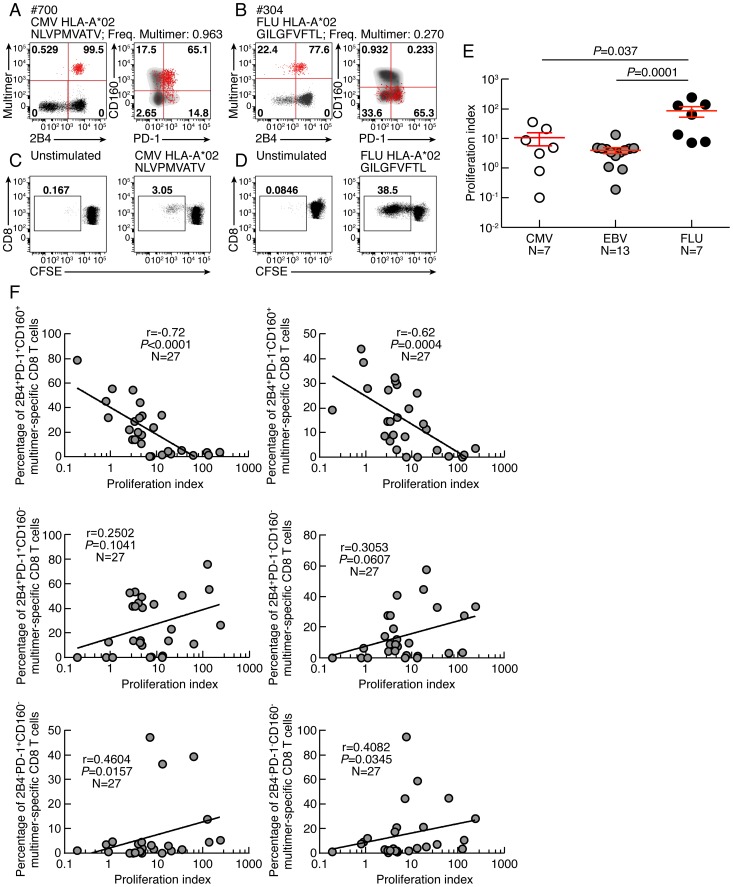
CD160, but not PD-1 and/or 2B4 expression inversely correlates with CD8 T-cell proliferative capacity. **(A–B)** Representative flow cytometric profiles of 2B4, PD-1 and CD160 expression of CMV (#700 CMV HLA-A*02 NLVPMVATV) and Flu (#304 FLU HLA-A*02 GILGFVFTL) specific CD8 T cells detected by multimer staining (red) as compared to total CD8 T cells (black/grey). **(C–D)** Representative CMV (#700 CMV HLA-A*02 NLVPMVATV) and Flu (#MP FLU HLA-A*02 GILGFVFTL) specific CD8 T-cell proliferation capacity assessed by CFSE based assay. **(E)** Proliferation index (CFSElow CD8 T-cell frequency/Multimer-specific CD8 T-cell frequency) CMV, EBV and FLU-specific CD8 T cells. Red bars correspond to mean ± SEM. **(F)** Correlations between proliferation index (CFSElow CD8 T-cell frequency/Multimer-specific CD8 T-cell frequency; x axes) and the virus-specific CD8 T-cell subsets distribution (percentage of Multimer-specific CD8 T cells expressing the different combinations of 2B4, PD1 and CD160; y axes). Statistical significance (*P* values) in panel **E** were obtained using One-way ANOVA (Kruskal-Wallis test) followed by a Student's t-test. *P* values in **F** were obtained using Spearman's rank correlations.

As mentioned above, CD8 T-cell proliferation capacity is one of the first function lost during CD8 T-cell functional impairment [11]–[14], [20]. The proliferation capacity of Flu, EBV and CMV-specific CD8 T cells was then assessed using the CFSE flow cytometry assay. To normalize according to the initial frequency of virus-specific CD8 T cells, virus-specific CD8 T-cell proliferation capacity was represented in proliferation index, defined as the ratio of the virus-specific CD8 T-cell proliferation capacity (percentage of CFSE low CD8 T cells) and the *ex vivo* frequency of virus-specific CD8 T cells (measured by MHC class I multimer staining). The representative flow cytometry profiles and the cumulative data showed that the proliferation index of Flu-specific CD8 T cells was significantly higher than that of EBV and CMV-specific CD8 T cells (Fig. 2 A–E). We then evaluated the potential association between the *ex vivo* expression pattern of co-inhibitory molecules expression and the proliferation index of virus-specific CD8 T cells. We showed that the *ex vivo* proportion of CD160, 2B4 but not PD-1 inversely correlated with the proliferation index of virus-specific CD8 T cells (r = −0.7605; *P*<0.0001, r = −0.5248; *P* = 0.0049, and r = 0.1002; *P* = 0.6192, respectively) (Fig. S3). We then assessed whether certain combinations of co-inhibitory molecules expression would be specifically associated with the proliferation index of virus-specific CD8 T cells. We showed that the *ex vivo* proportion of 2B4^+^CD160^+^PD-1^+^ or 2B4^+^CD160^+^PD-1^−^ virus-specific CD8 T cells inversely correlated with the proliferation index of virus-specific CD8 T cells (r = −0.72; *P*<0.0001 and r = −0.62; *P* = 0.0004, respectively) (Fig. 2F). In addition, the proliferation index of virus-specific CD8 T cells did not inversely correlate with the proportion of CD160^−^ CD8 T-cell populations (Fig. 2F). These data suggest that the proliferation capacity of virus-specific CD8 T cells may be negatively regulated by CD160 expression independently of PD-1 expression.

### Proliferative capacity virus-specific CD8 T-cell populations is influenced by co-inhibitory molecule expression

**Figure 3:**
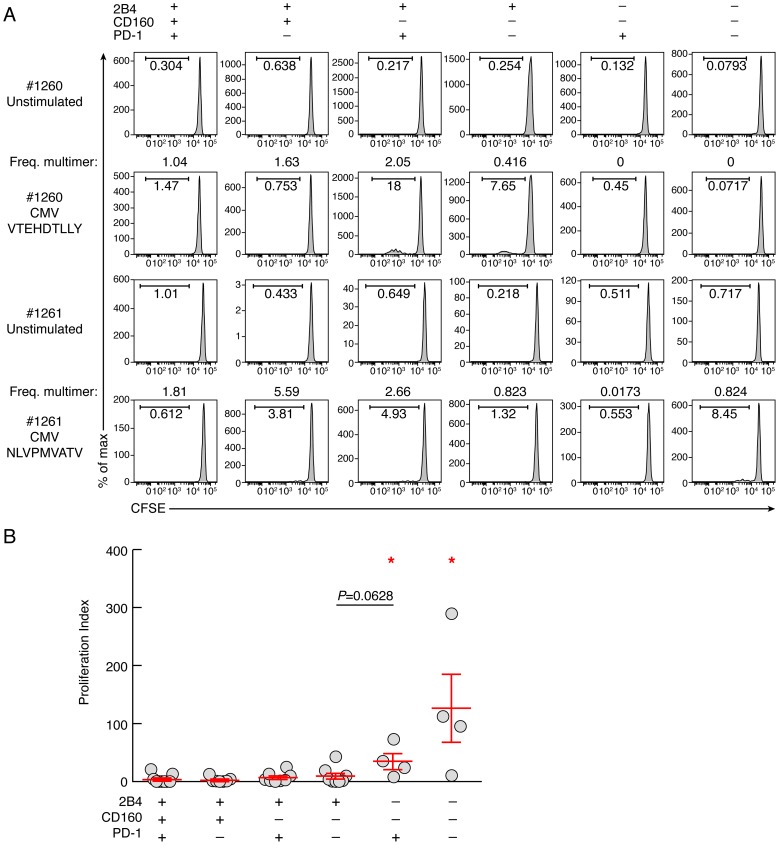
Proliferative capacity virus-specific CD8 T-cell populations is influenced by co-inhibitory molecule expression. CD8 T-cell populations were sorted on the basis of 2B4, PD-1 and CD160 expression, labeled with CFSE and stimulated with (viral) peptides in the presence of autologous irradiated CD8-depleted PBMCs (ratio CD8/feeder cells 1∶10) for 6 days (n = 11). **(A)** Representative CMV (#1260 CMV HLA-A*01 VTEHDTLLY) and CMV (#1261 CMV HLA-A*02 NLVPMVATV) specific CD8 T-cell proliferation capacity assessed by CFSE based assay. **(B)** Proliferation index (CFSElow CD8 T-cell frequency/Multimer-specific CD8 T-cell frequency) of virus-specific CD8 T cells. Red bars correspond to mean ± SEM. Red stars indicate statistical significance (*P*<0.05). Statistical significance (*P* values) in panel B was obtained using One-way ANOVA (Kruskal-Wallis test) followed by a Student's t-test.

To determine the impact of co-inhibitory signals on virus-specific CD8 T-cell proliferation capacity, CD8 T-cell populations were sorted based on PD-1, 2B4 and CD160 expression. Of note, naïve CD8 T cells (CD45RA^+^CCR7^+^) were excluded from the analysis. Cell populations were then stimulated with (viral) peptides in the presence of antigen presenting cells (CD8-depleted PBMCs) for 6 days (Fig. 3). The proliferation capacity of Flu, EBV and CMV-specific CD8 T cells was then assessed using the CFSE flow cytometry assay and normalized according to the initial frequency of virus-specific CD8 T cells within each CD8 T-cell population (*i.e.* 2B4^+^CD160^+^PD-1^+^, 2B4^+^CD160^+^PD-1^−^, 2B4^+^CD160^−^PD-1^+^, 2B4^+^CD160^−^PD-1^−^, 2B4^−^CD160^−^PD-1^+^ and 2B4^−^CD160^−^PD-1^−^ CD8 T-cell populations). The representative flow cytometric profiles as well as the cumulative data show that the proliferation capacity of virus-specific 2B4^+^CD160^+^PD-1^+^, 2B4^+^CD160^+^PD-1^−^, 2B4^+^CD160^−^PD-1^+^, 2B4^+^CD160^−^PD-1^−^ CD8 T-cell populations was significantly reduced as compared to 2B4^−^CD160^−^PD-1^+^ and 2B4^−^CD160^−^PD-1^−^ CD8 T-cell populations (*P*<0.05) (Fig. 3A–B). Of note, the proliferation capacity of 2B4^+^CD160^−^PD-1^−^ and 2B4^−^CD160^+^PD-1^+^ CD8 T cells was not significantly different (*P* = 0.0628) (Fig. 3B). These data suggest that the proliferation capacity of virus-specific CD8 T cells is influenced by co-inhibitory molecule expression.

### CD8 T-cell populations expressing CD160 harbor reduced proliferative capacity, independently of PD-1 expression

**Figure 4:**
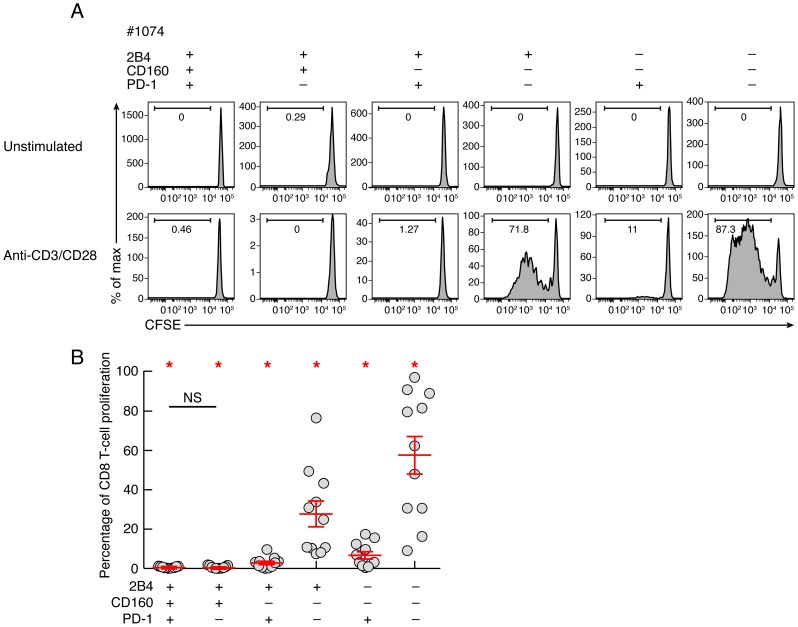
Intrinsic proliferation capacity of sorted CD8 T-cell populations expressing the different combinations of 2B4, CD160 and PD-1. CD8 T-cell populations were sorted on the basis of 2B4, PD-1 and CD160 expression, labeled with CFSE and stimulated in anti-CD3 and anti-CD28 MAbs coated plate for 6 days (n = 11). The percentage of proliferating CD8 T cells (CFSE low) was assessed by flow cytometry at day 6. **(A)** Representative flow cytometric profiles of proliferating CD8 T-cell populations following anti-CD3/CD8 MAbs stimulation. Unstimulated cells (negative control) are also shown. **(B)** Percentage of CD8 T-cell proliferation of each CD8 T-cell subset upon 6 days stimulation with a-CD3/CD28 coated mAbs. Red bars correspond to mean ± SEM. Red stars indicate statistical significance (*P*<0.05). NS: not significant. Statistical significance (*P* values) in panels A and B were obtained using One-way ANOVA (Kruskal-Wallis test) followed by a paired Student's t-test.

To determine the intrinsic CD8 T-cell proliferation capacity, CD8 T-cell populations were sorted based on PD-1, 2B4 and CD160 expression. Of note, naïve CD8 T cells (CD45RA^+^CCR7^+^) were excluded from the analysis. Cell populations were then stimulated with coated anti-CD3 and anti-CD28 MAbs for 6 days, and the proliferation capacity was assessed using CFSE flow cytometry based assay (Fig. 4). The representative flow cytometric profiles and the cumulative data showed that the proliferation capacity of CD160 and/or PD-1-expressing CD8 T cells was significantly reduced as compared to CD160 and PD-1 negative CD8 T-cell populations (*P*<0.05) (Fig. 4). Interestingly, the proliferation capacity of 2B4^+^CD160^+^PD-1^+^ and 2B4^+^CD160^+^PD-1^−^ CD8 T cells was not significantly different (*P* = 0.6786), suggesting that CD160-expessing CD8 T cells are endowed with reduced proliferation capacity, independently of PD-1 expression.

### CD160 but not PD-1 expression is strongly associated with reduced IL-2 production

**Figure 5:**
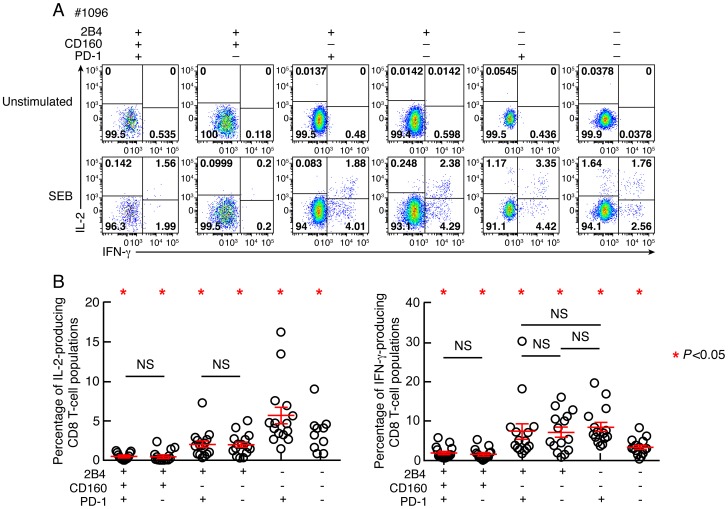
Relative capacity of CD8 T-cell subsets defined by 2B4, CD160 and PD-1 expression to produce IL-2 and IFN-γ. CD8 T-cell responses were analyzed within six CD8 T-cell subsets defined by 2B4, CD160 and/or PD-1 expression. **(A)** Representative flow cytometric profiles of CD8 T cells producing IL-2 and/or IFN-γ following stimulation of total blood mononuclear cells with SEB in Subject #1096. Unstimulated cells (negative control) are also shown. **(B)** Frequencies of IL-2 and IFN-γ-producing CD8 T-cell populations. Red bars correspond to mean ± SEM. Red stars indicate statistical significance (*P*<0.05). NS: not significant. Statistical significance (*P* values) in panels **A** and **B** were obtained using One-way ANOVA (Kruskal-Wallis test) followed by a paired Student's t-test.

In chronic virus infections the loss of CD8 T-cell proliferative capacity is commonly associated with a progressive reduction of IL-2 production [15], [16]. Production of IFN-γ is affected generally at advanced stages of T-cell exhaustion [15], [16]. IL-2 and IFN-γ production were then evaluated by flow cytometry in memory CD8 T-cell populations defined by PD-1, 2B4 and CD160 expression. Of note, the naïve CD8 T-cell population (CD45RA^+^CCR7^+^) was excluded from this analysis. The representative flow cytometric profiles as well as the cumulative data showed that the frequencies of IL-2 and IFN-γ-producing cells were significantly reduced in CD160^+^ CD8 T-cell populations as compared to CD160^−^ CD8 T-cell populations (*P*<0.05) (Fig. 5A–C). The frequencies of both IL-2 and IFN-γ-producing cells were not significantly different between 2B4^+^CD160^+^PD-1^+^ and 2B4^+^CD160^+^PD-1^−^ CD8 T-cell populations (*P* = 0.998 and *P* = 0.470, respectively) and between 2B4^+^CD160^−^PD-1^+^ and 2B4^+^CD160^−^PD-1^−^ CD8 T-cell populations (*P* = 0.888 and *P* = 0.908, respectively) (Fig. 5A–C). In addition, 2B4^−^CD160^−^PD-1^+^ CD8 T-cell populations contained higher frequencies of IL-2-producing cells than any other CD8 T-cell population (*P*<0.05) (Fig. 5A–C), demonstrating that CD160 and not PD-1 expression on memory CD8 T cells is strongly associated with reduced IL-2 and IFN-γ production capacity.

### Co-inhibitory molecule expression is associated with the differentiation state

**Figure 6:**
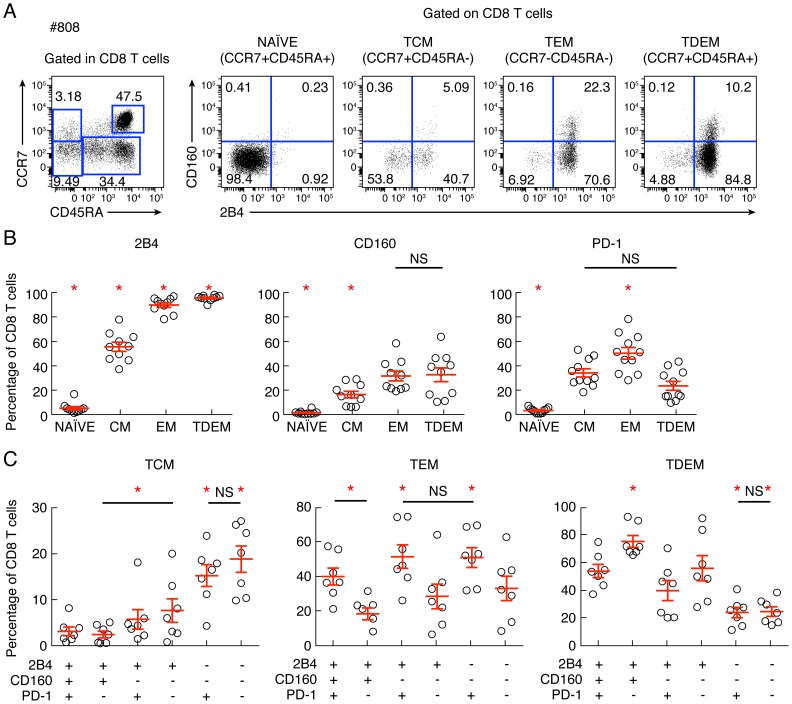
Combined assessment of co-inhibitory molecule expression and differentiation state. CD8 T-cell differentiation and CD8 T-cell expression of 2B4, CD160 and/or PD-1 were analyzed. **(A)** Representative example of flow cytometric profile of CD8 T-cell subsets defined by CCR7 and CD45RA expressing 2B4 and/or CD160. **(B)** Cumulative analyses (n = 10) of co-inhibitory molecules expression within 3 distinct memory CD8 T-cell subsets. **(C)** Cumulative analyses (n = 10) of the differentiation within 6 distinct CD8 T-cell population defined by 2B4, PD-1 and CD160 expression. NS: not significant. Statistical significance (*P* values) was obtained using One-way ANOVA (Kruskal-Wallis test) followed by a paired Student's t-test. Red bars correspond to mean ± SEM. Red stars indicate statistical significance (*P*<0.05).

Reduced IL-2 production capacity is commonly associated with increased differentiation state [21]. In this context, we assessed the potential association between co-inhibitory molecule expression and differentiation state. To that purpose, the differentiation state was evaluated using the expression of CD45RA and CCR7. The representative flow cytometric profiles as well as the cumulative data showed that 2B4, CD160 and PD-1 expression significantly increased with the differentiation state, confirming the recent findings from Legat and colleagues [22] (Fig. 6A–B). However, in-depth analyses showed that CD8 T-cell populations defined by 2B4, CD160 and/or PD-1 expression were heterogeneously distributed among the distinct differentiated CD8 T-cell subsets (Fig. 6C). Indeed, the central memory compartment (CM; CD45RA^−^CCR7^+^) was significantly enriched in 2B4^−^CD160^−^PD-1^−^ and 2B4^−^CD160^−^PD-1^+^ CD8 T-cell populations, the effector memory compartment (EM; CD45RA^−^CCR7^−^) was significantly enriched in PD-1^+^ CD8 T-cell populations (2B4^+^CD160^+^PD-1^+^, 2B4^+^CD160^−^PD-1^+^ and 2B4^−^CD160^−^PD-1^+^ CD8 T-cell populations) and the terminally differentiated effector memory compartment (TDEM; CD45RA^+^CCR7^−^) was significantly enriched in the 2B4^+^CD160^+^PD-1^−^ CD8 T-cell population (*P*<0,05) (Fig. 6C). These data suggest that co-inhibitory molecule expression is associated with differentiation state. However in depth analyses further indicated that significant differences are present within each CD8 T-cell populations, particularly among PD-1 and/or CD160-expressing populations.

### CD160 but not PD-1 expression is strongly associated with reduced perforin expression

**Figure 7:**
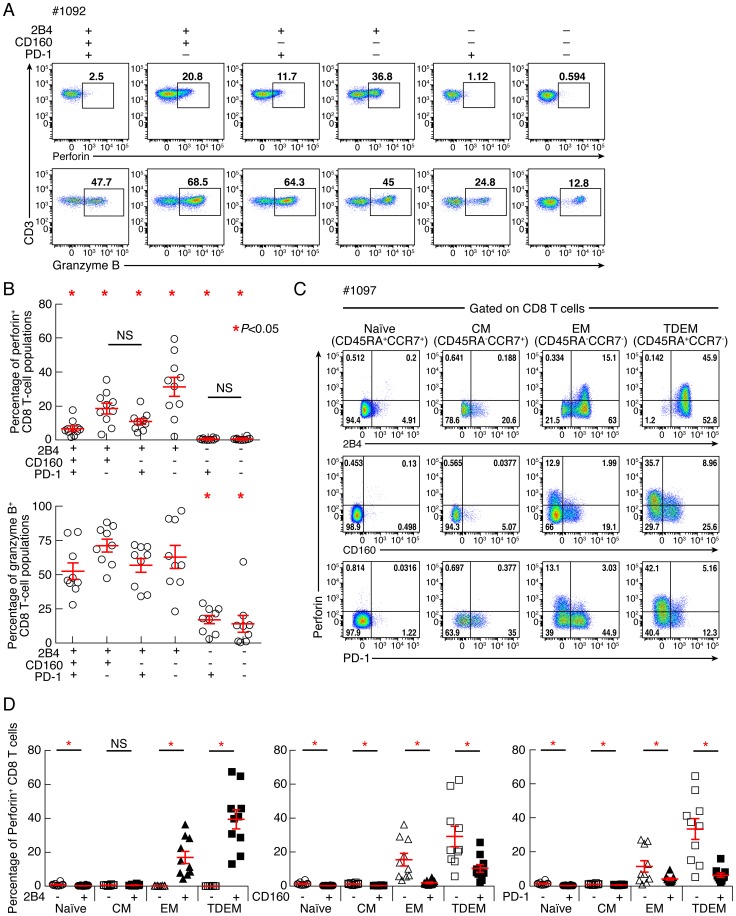
Expression levels of perforin and granzyme B in CD8 T-cell subsets defined by the expression of 2B4, CD160 and PD-1. CD8 T-cell responses were analyzed within six CD8 T-cell subsets defined by 2B4, CD160 and/or PD-1 expression. **(A)** Representative flow cytometric profiles of CD8 T cells expressing perforin and/or granzyme B *ex vivo*. **(B)** Frequencies of CD8 T cells expressing perforin and granzyme B. **(C)** Representative flow cytometric profiles of differentiated CD8 T-cell subsets defined by the CCR7 and CD45RA expressing perforin, 2B4, CD160 and PD-1. (D) Cumulative analyses (n = 10) representing the expression of perforin differentiated CD8 T-cell subsets in function of CD160 expression. Red bars correspond to mean ± SEM. Red stars indicate statistical significance (*P*<0.05). NS: not significant. Statistical significance (*P* values) in panels **B** and **D** were obtained using One-way ANOVA (Kruskal-Wallis test) followed by a paired Student's t-test.

Since perforin expression is the hallmark of differentiated CD8 T cells [21], we then assessed whether 2B4^+^CD160^+^PD-1^+^ and 2B4^+^CD160^+^PD-1^−^ CD8 T-cell populations were enriched in perforin or granzyme B. Indeed, cytotoxic CD8 T cells exert their antiviral activity primarily through the secretion of cytotoxic granules containing perforin and granzymes [23], [24]. Therefore, the expression of perforin and granzyme B was evaluated in CD8 T-cell populations defined by the expression of PD-1, 2B4 and/or CD160. Of note, the naïve CD8 T-cell population (CD45RA^+^CCR7^+^) was excluded from this analysis. As shown in the representative flow cytometric profiles and the cumulative data perforin and granzyme B were significantly enriched in 2B4^+^ CD8 T-cell population as compared to 2B4^−^ CD8 T-cell populations (*P*<0.05) (Fig. 7A–B) consistently with a previous study [25]. Within 2B4^+^ CD8 T-cell populations, perforin expression was not significantly different between 2B4^+^PD-1^−^CD160^+^ and 2B4^+^PD-1^+^CD160^−^ CD8 T-cell populations (*P* = 0.1596). However, perforin expression was significantly reduced in 2B4^+^PD-1^+^CD160^+^, 2B4^+^PD-1^−^CD160^+^ and 2B4^+^PD-1^+^CD160^−^ CD8 T-cell populations as compared to the 2B4^+^PD-1^−^CD160^−^ CD8 T-cell population (*P* = 0.0014, *P* = 0.0047 and *P* = 0.0039, respectively). Interestingly, perforin expression was significantly reduced in 2B4^+^PD-1^+^CD160^+^ as compared to 2B4^+^PD-1^−^CD160^+^ and 2B4^+^PD-1^+^CD160^−^ CD8 T-cell populations (*P* = 0.0092 and *P* = 0.0201, respectively). Finally, the potential association between perforin and co-inhibitory molecule expression was assessed within each differentiated CD8 T-cell subset *i.e.* naïve, central memory, effector memory and terminally differentiated effector memory. The representative flow cytometric profiles and the cumulative data showed that perforin expression was significantly enriched within 2B4-expressing CD8 T cells in both EM (*P* = 0.0011) and TDEM (*P*<0.0001) (Fig. 7C–D). Interestingly, perforin expression was significantly reduced in CD160-expressing CD8 T cells in both EM (*P* = 0.0063) and TDEM (*P* = 0.026) and in PD-1-expressing CD8 T cells in both EM (*P* = 0.0391) and TDEM (*P* = 0.00031) (Fig. 7C–D). Taken together, these data demonstrate that CD8 T-cell populations expressing CD160 and/or PD-1 harbored reduced perforin expression, independently of the differentiation state (Fig. 7C–D). Of note, the percentage of expression of EOMES, T-bet and CD57 (immunosenescence [26]) was not significantly different (*P*>0.05) between 2B4^+^CD160^+^PD-1^−^ and 2B4^+^CD160^+^PD-1^+^ CD8 T-cell populations (Fig. S4), suggesting that 2B4^+^CD160^+^PD-1^+^ CD8 T-cell population was not more immunosenescent than 2B4^+^CD160^+^PD-1^−^ CD8 T-cell population. Taken together, these data demonstrate that the expression of CD160 and/or PD-1 on 2B4^+^ CD8 T cells is associated with a reduced capacity to express perforin as compared to the 2B4^+^CD160^−^PD-1^−^ CD8 T-cell population, independently on the differentiation state.

### CD160 blockade significantly increases virus-specific CD8 T-cell proliferation

**Figure 8:**
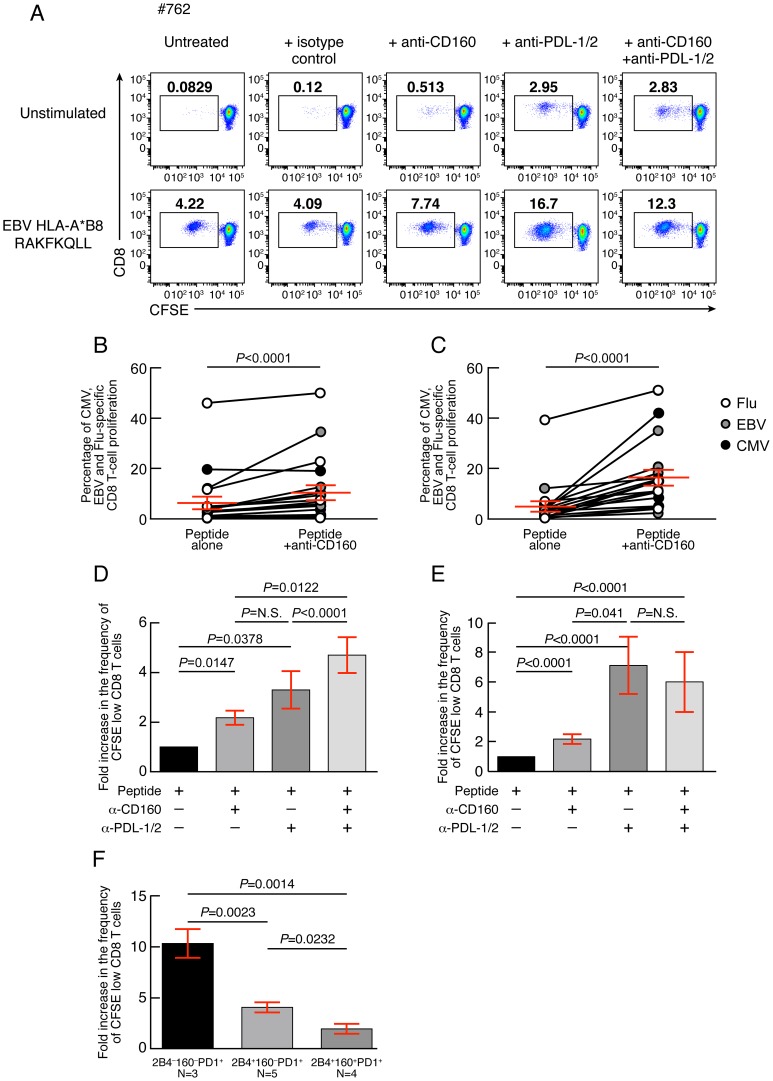
CD160 blockade significantly increases virus-specific CD8 T-cell proliferation. **(A)** Representative example of EBV-specific CD8 T-cell proliferation (#675 EBV HLA-B*08 RAKFKQLL) in presence or in absence of anti-CD160 mAbs and/or anti-PDL-1/2 mAbs assessed by CFSE-based assay. **(B)** Percentage of CMV, EBV and Flu-specific CD8 T-cell proliferation in presence or not of anti-CD160 mAb (N = 21). **(C)** Percentage of CMV, EBV and Flu-specific CD8 T-cell proliferation in presence or not of anti-PDL-1/2 mAb (N = 21). **(D)** Fold increase in the frequency of CFSE low CD8 T cells in presence of anti-CD160 and/or anti-PDL-1/2 mAb as compared to untreated virus-specific CD8 T cells from virus-specific CD8 T-cell populations harboring both 2B4^+^CD160^−^PD-1^+^ and 2B4^+^CD160^+^PD-1^−^ CD8 T-cell populations (2B4^+^CD160^−^PD-1^+^ and 2B4^+^CD160^+^PD-1^−^ virus-specific CD8 T cells >10%) (N = 5) or **(E)** from virus-specific CD8 T-cell populations containing the highest proportion of 2B4^+^CD160^+^PD-1^+^ CD8 T-cell population (2B4^+^CD160^+^PD-1^+^ virus-specific CD8 T cells >30%) (N = 6). **(F)** Fold increase in the frequency of CFSE low CD8 T cells in presence of anti-PDL-1/2 mAb on virus-specific CD8 T cells dominated by 2B4^−^CD160^−^PD-1^+^, 2B4^+^CD160^−^PD-1^+^ or 2B4^+^CD160^+^PD-1^+^ CD8 T-cell populations. Red bars correspond to mean ± SEM. Statistical significance (*P* values) were obtained using One-way ANOVA (Kruskal-Wallis test) (panel **D–F**) followed by a paired Student's t-test (panels **B–C**), Wilcoxon Signed Rank test (**D–E**), or unpaired Student's t-test (panels **F**).

In order to assess the relative influence of CD160 signaling as compared to PD-1 signaling on the virus-specific CD8 T-cell proliferation capacity, the proliferation capacity of CMV, EBV and Flu-specific CD8 T cells was evaluated in the presence or in the absence of 1) blocking anti-CD160 monoclonal antibodies (mAbs), 2) blocking anti-PD-L1/2 mAbs, 3) combined blocking anti-CD160 and anti-PD-L1/2 mAbs and 4) isotype controls. Of note, anti-CD160 MAbs did not show any agonistic potential either in absence or in presence of TCR signal (data not shown), as assessed by CD69, CD107a and BCL-2 expression or TNF-α and IFN-γ production (data not shown). The representative flow cytometric profiles showed that both anti-CD160 and anti-PD-1 mAb treatments individually increased the proliferation capacity of EBV-specific CD8 T cells (Fig. 8A). In addition, the cumulative data showed that both anti-CD160 and anti-PD-1 mAb treatments individually increased the proliferation capacity of CMV, EBV and Flu-specific CD8 T cells (2.06 fold increase; *P*<0.0001; and 7.6 fold increase; *P*<0.0001, respectively) (Fig. 8B–C). The effects of the combined blockade of CD160 and PD-1 signaling pathways as compared to PD-1 or CD160 signaling blockade alone on CD8 T-cell proliferation were assessed on virus-specific CD8 T-cell populations harboring 1) both 2B4^+^CD160^−^PD-1^+^ and 2B4^+^CD160^+^PD-1^−^ CD8 T-cell populations (2B4^+^CD160^−^PD-1^+^ and 2B4^+^CD160^+^PD-1^−^ virus-specific CD8 T cells >10%; N = 5) (Fig. 8D) and 2) containing the highest proportion of 2B4^+^CD160^+^PD-1^+^ CD8 T-cell population (2B4^+^CD160^+^PD-1^+^ virus-specific CD8 T cells >30%; N = 6) (Fig. 8E). The cumulative data show that the combined blockade of CD160 and PD-1 signaling pathways significantly increased CD8 T-cell proliferation as compared to PD-1 or CD160 signaling blockade alone, in virus-specific CD8 T cells containing both 2B4^+^CD160^−^PD-1^+^ and 2B4^+^CD160^+^PD-1^−^ CD8 T-cell populations (2B4^+^CD160^−^PD-1^+^ and 2B4^+^CD160^+^PD-1^−^ virus-specific CD8 T cells >10%; N = 5) (*P*<0.05) (Fig. 8D). However, consistently with the study by Peretz *et al.* [18], the combined blockade of CD160 and PD-1 signaling pathways did not significantly increase CD8 T-cell proliferation as compared to PD-1 signaling blockade alone, in virus-specific CD8 T cells containing the highest proportion of 2B4^+^CD160^+^PD-1^+^ CD8 T-cell population (2B4^+^CD160^+^PD-1^+^ virus-specific CD8 T cells >30%; N = 6) (P>0.05) (Fig. 8E). In addition, PD-1 signaling blockade was significantly more potent on virus-specific CD8 T cells with dominant 2B4^−^CD160^−^PD-1^+^ CD8 T-cell population as compared to virus-specific CD8 T cells with dominant 2B4^+^CD160^−^PD-1^+^ or 2B4^+^CD160^+^PD-1^+^ CD8 T-cell populations (10.36 *versus* 4.06 and 1.95 fold increase; *P* = 0.0014 and *P* = 0.0023, respectively) (Fig. 8F). Of note, PD-1 mean fluorescent intensity (MFI) was not significantly different between 2B4^−^CD160^−^PD-1^+^ and 2B4^+^CD160^+^PD-1^+^ CD8 T-cell populations (Fig. S5). Interestingly, however, the 2B4^+^CD160^+^PD-1^−^ CD8 T-cell population expressed the highest levels of CD160 (MFI) and 2B4 (MFI) as compared to any other CD8 T-cell population (Fig. S5). Taken together, these data demonstrate that a) CD160 and PD-1 signaling negatively regulate TCR-mediated CD8 T-cell signaling, b) the functional restoration induced by the blockade of multiple co-inhibitory molecules may be incomplete, and c) the cells expressing several co-inhibitory molecules are more profoundly impaired than cells expressing only one co-inhibitory molecule.

### Restoration of CD8 T-cell proliferation by CD160/CD160-ligand blockade directly correlates with the level of the *ex vivo* CD160 expression

**Figure 9:**
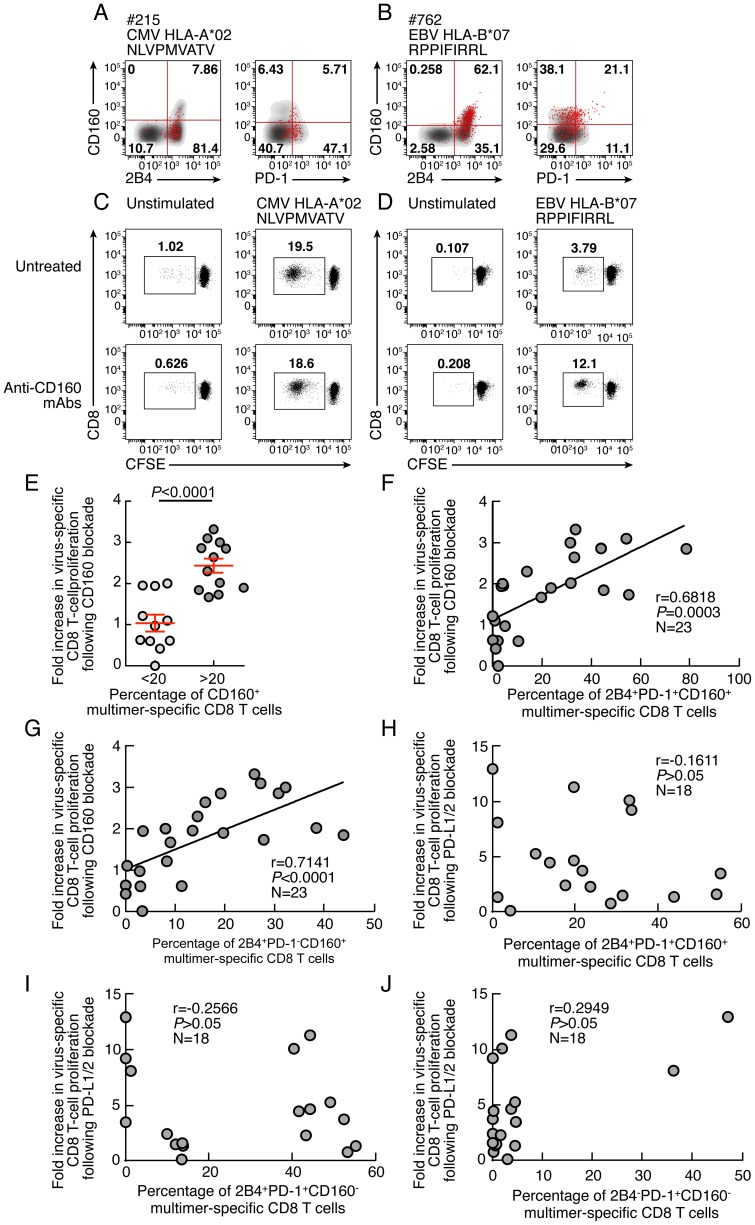
Restoration of CD8 T-cell proliferation by CD160/CD160-ligand blockade directly correlates with the level of the *ex vivo* CD160 expression. **(A–B)** Representative flow cytometric profile of 2B4, CD160 and PD-1 expression of CMV (#215 CMV HLA-A*02 NLVPMVATV) and EBV (#762 EBV HLA-B*07 RPPIFIRRL)-specific CD8 T cells detected by multimer staining (red) compared with total CD8 T cells (black/grey). **(C–D)** Representative examples of CMV (#215 CMV HLA-A*02 NLVPMVATV) and EBV (#762 EBV HLA-B*07 RPPIFIRRL)-specific CD8 T-cell proliferation in presence or not of anti-CD160 mAbs assessed by CFSE-based assay. **(E)** Impact of CD160 expression on the restoration of CD8 T-cell proliferation by CD160/CD160-ligand blockade. **(F–G)** Correlation between fold increase in proliferation capacity (y axes) upon CD160 blockage and percentage of 2B4^+^CD160^+^PD-1^+^ or 2B4^+^CD160^+^PD-1^−^ virus-specific CD8 T cells. **(H–J)** Correlation between fold increase in proliferation capacity (y axes) upon PDL-1/2 blockage and percentage of 2B4^+^CD160^+^PD-1^+^, 2B4^+^CD160^−^PD-1^+^ or 2B4^−^CD160^−^PD-1^+^ virus-specific CD8 T cells (x axes). Statistical significance (*P* values) in **B**–**I** were obtained using Spearman's rank correlations.

Since the expression level of co-inhibitory molecules on virus-specific CD8 T cells is highly heterogeneous (Fig. 1), the influence of CD160 and PD-1 signaling blockade was evaluated with regard to the *ex vivo* expression levels of CD160 and PD-1. The representative flow cytometric profiles as well as the cumulative data showed that CD160 signaling blockade was significantly more potent in cells harboring high level of CD160 (percentage of virus-specific CD8 expressing more than 20% of CD160) versus cells harboring low level of CD160 (percentage of virus-specific CD8 expressing less than 20% of CD160) (P<0.0001) (Fig. 9A–E). In addition, the degree of restoration of virus-specific CD8 T-cell proliferation capacity induced by CD160 blockade directly correlated with the *ex vivo* proportion of 2B4^+^CD160^+^PD-1^+^ or 2B4^+^CD160^+^PD-1^−^ virus-specific CD8 T cells (r = 0.6818; *P* = 0.0003 and r = 0.7141; *P*<0.0001, respectively) (Fig. 9F–G). Interestingly, the effect of the blockade of PD-1/PD-L1/2 pathway on the degree of the restoration of virus-specific CD8 T-cell proliferation capacity was not associated with the *ex vivo* proportion of 2B4^+^CD160^+^PD-1^+^, 2B4^+^CD160^−^PD-1^+^ or 2B4^−^CD160^−^PD-1^+^ virus-specific CD8 T cells (r = −0.1611; *P*>0.05, and r = −0.2566; *P*>0.05 and r = 0.2949; *P*>0.05, respectively) (Fig. 9H–J).

### CD160 expression is not up-regulated upon T-cell activation or proliferation

**Figure 10:**
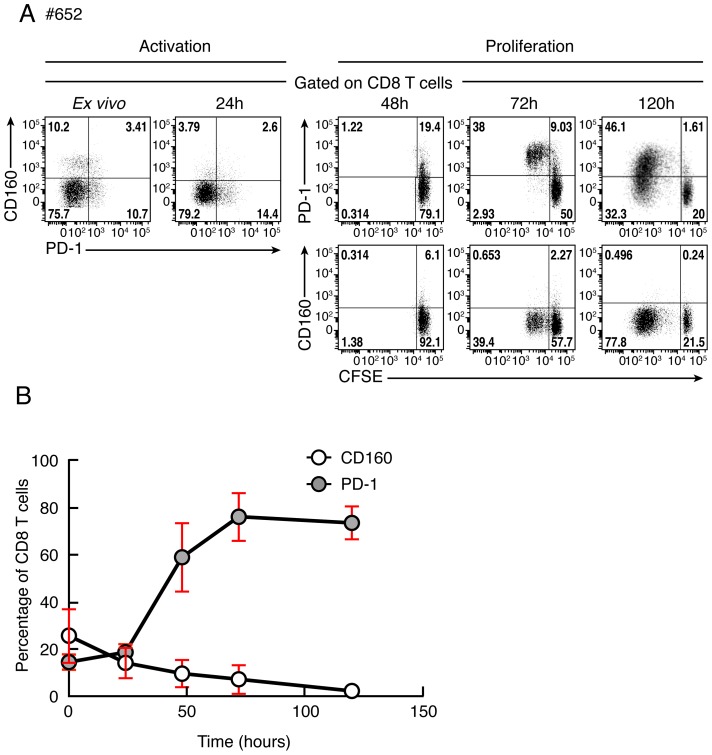
Kinetic of CD160 or PD-1 expression on CD8 T cells stimulated upon T-cell stimulation. **(A)** Representative flow cytometric profile of CD160 and PD-1 expression on CD8 T cells after 0, 24, 48, 72 and 120 hours of stimulation with anti-CD3/anti-CD28 magnetic beads. CD8 T-cell proliferation was also measured by CFSE-based assay at 48, 72 and 120 hours after stimulation. **(B)** Cumulative data of CD160 or PD-1-expressing CD8 T-cell frequencies at distinct time points after stimulation. Red bars correspond to mean ± SEM.

Previous studies have indicated that PD-1 expression increased following T-cell stimulation and activation [27]. However, the regulation of CD160 expression following T-cell activation or proliferation remains unclear. We then evaluated the expression levels of PD-1 and CD160 co-inhibitory molecules following TCR stimulation using flow cytometry. Briefly, cells were stained with CFSE, stimulated with anti-CD3 plus anti-CD28 Abs and the expression levels of PD-1 and CD160 were assessed by flow cytometry at day 0, 1, 2, 3 and 5. Representative flow cytometric profiles as well as cumulative data showed that CD160 expression was down-regulated in CD8 T cells upon T-cell activation and proliferation, while PD-1 expression increased (Fig. 10A–B).

## Discussion

Several studies have demonstrated that the increased expression of co-inhibitory molecules including PD-1, 2B4 and CD160 during chronic viral infections is associated with CD8 T-cell dysfunction [2], [17], [18], [28]. However, the relative contribution of individual co-inhibitory molecules in the T-cell functional impairment remains unclear.

In the present study, we have evaluated the impact of individual co-inhibitory molecules expression such as 2B4, PD-1 and CD160 on virus-specific CD8 T-cell functions. To address this issue, we have performed both phenotypic and functional characterization of Flu, EBV and CMV-specific CD8 T cells in healthy individuals. We show that EBV and CMV-specific CD8 T cells contained higher frequency of cells co-expressing 2B4, CD160 and PD-1 in different combinations while Flu-specific CD8 T cells had higher frequencies of triple negative, single PD-1 or dual PD-1/2B4 CD8 T-cell populations. In particular, the percentage of CD160 expression was significantly lower in Flu-specific CD8 T cells than in CMV or EBV-specific CD8 T cells. Interestingly, the level of total PD-1 was not significantly different in Flu, EBV or CMV-specific CD8 T cells. These data suggest that CD160 expression in virus-specific CD8 T cells is associated with chronic virus infections such as EBV and CMV. Of note, co-inhibitory molecule expression on virus-specific CD8 T cells are likely influenced by the pathogen characteristics, the type of viral infection (cleared *versus* chronic viral infections), the cytokine milieu, the nature of the infected cell population.

Proliferation capacity is one of the first CD8 T-cell functions lost during the progression to CD8 T-cell exhaustion [11]–[14], [20]. In this regard, we assessed which pattern of co-inhibitory molecules expression had the greater impact on the proliferation capacity of virus-specific CD8 T cells. We showed that the proportion of total CD160 expression and in particular of 2B4^+^CD160^+^PD-1^+^ or 2B4^+^CD160^+^PD-1^−^ CD8 T-cell populations inversely correlated with the proliferation capacity thus suggesting that CD160 expression was associated with reduced CD8 T-cell proliferation capacity independently of PD-1 expression. The reduced proliferation capacity of sorted virus-specific 2B4^+^CD160^+^PD-1^+^, 2B4^+^CD160^+^PD-1^−^, 2B4^+^CD160^−^PD-1^+^, 2B4^+^CD160^−^PD-1^−^ CD8 T-cell populations as compared to 2B4^−^CD160^−^PD-1^+^ and 2B4^−^CD160^−^PD-1^−^ CD8 T-cell populations suggest that the proliferation capacity CD8 T cells is influenced by co-inhibitory molecule expression.

The intrinsic proliferation capacity of the different CD8 T-cell populations was then evaluated and showed that CD8 T cells expressing CD160 and/or PD-1 were endowed with reduced proliferation capacity as compared to CD160 and PD-1 negative CD8 T-cell populations. Interestingly, the proliferation capacity of 2B4^+^CD160^+^PD-1^+^ and 2B4^+^CD160^+^PD-1^−^ CD8 T cells was not significantly different, suggesting that CD160 expressing CD8 T cells are endowed with reduced proliferation capacity, independently of PD-1 expression.

We then showed that 2B4^+^CD160^+^PD-1^+^ or 2B4^+^CD160^+^PD-1^−^ CD8 T-cell populations also had a reduced capacity to produce IL-2, IFN-γ and perforin as compared to CD160^−^ CD8 T-cell populations demonstrating that CD160^+^ CD8 T-cell populations were more functionally impaired than CD160^−^ CD8 T-cell populations independently of PD-1 expression. Since reduced IL-2 production capacity is commonly associated with increased differentiation state [21], the differentiation state was evaluated together with the expression of co-inhibitory molecules. We showed that co-inhibitory molecule expression is associated with differentiation state [22]. However in depth analyses indicated that significant differences are present within each CD8 T-cell populations, particularly among PD-1 and/or CD160-expressing populations and suggested that CD8 T-cell functions might be modulated by co-inhibitory molecule expression.

The functions of CD160 are complex, and still remain under debate, with potentially various roles *i.e.* either stimulatory or inhibitory, depending on the cell type, the expression of the ligands (HVEM or MHC class I) [29]–[32] and the fact that CD160 exist in two isoforms (spliced variants) with and without transmembrane (TM) domain [33], [34]. In order to determine whether CD160/CD160-ligand interactions could negatively regulate CD8 T-cell signaling, virus-specific CD8 T-cell proliferation was assessed in the presence or in the absence of mAbs that block CD160/CD160-ligand signaling. Since two molecules have been shown to interact with CD160 *i.e.* HVEM (member of the TNF receptor superfamily) and MHC class I molecules [29]–[32], blocking anti-CD160 Abs that prevent both possible interactions were used [31], [35]. Of note, HVEM is broadly distributed and expressed in hematopoietic and non-hematopoietic cells [36]. The ability of anti-CD160 Abs to increase virus-specific CD8 T-cell proliferation demonstrated that CD160 signaling negatively regulates TCR-mediated CD8 T-cell signaling. Interestingly, the level of restoration of virus-specific CD8 T-cell proliferation capacity induced by CD160 blockade was significantly more potent in cells harboring high level of CD160 and directly correlated with the *ex vivo* proportion of CD160 expression independently of PD-1 expression. These results suggested that 2B4^+^CD160^+^PD-1^+^ and 2B4^+^CD160^+^PD-1^−^ CD8 T-cell populations were both rescued by CD160/CD160-ligands signaling blockade.

We then showed that 2B4^+^CD160^+^PD-1^+^, 2B4^+^CD160^+^PD-1^−^ and 2B4^+^CD160^−^PD-1^+^ CD8 T-cell populations had reduced expression of perforin as compared to 2B4^+^CD160^−^PD-1^−^ CD8 T-cell population, suggesting that the expression of CD160 and/or PD-1 on 2B4^+^ CD8 T cells is associated with a reduced capacity to express perforin. Interestingly, CD160 signaling is commonly associated with enhanced NK-cell cytotoxic activity [30]–[32] suggesting that CD160 might have distinct functions depending upon the cell subset considered. Indeed, CD160 is a GPI-anchored protein lacking both transmembrane (TM) and intracytoplasmic domains [33], [34]). Therefore, the signaling cascade(s) triggered following CD160/CD160-ligand interactions might depend on the cell-type, the ligand (either HVEM or MHC class I molecules), the density of CD160 expression, the potential co expression with CD160-TM and on the downstream adaptor proteins and/or signaling molecules, which are still not fully characterized [37], [38]. We hypothesize that in CD8 T cells, CD160 might be associated with a specific phosphatase that might reduce the signal triggered following TCR/co-stimulatory receptors engagement. In addition, CD160 exists in two isoforms (spliced variants) with and without TM domain [33], [34]. While NK cells can express both isoforms *i.e.* CD160 and CD160 TM, that are modulated following IL-15 stimulation [34], CD8 T cells do not express CD160 TM directly *ex vivo* nor following IL-15 or TCR stimulation ([34] and Fig. S6). This feature may explain the dichotomic role of CD160 *i.e.* involved in CD8 T-cell negative regulation and NK-cell cytotoxic activity [17], [18], [30]–[32].

As previously demonstrated PD-1 signaling blockade by anti-PD-L1/2 Abs significantly increased virus-specific CD8 T-cell proliferation [12], [13], [17]. However, the PD-1 signaling blockade was significantly more potent on virus-specific CD8 T cells containing predominant 2B4^−^CD160^−^PD-1^+^ CD8 T-cell population as compared to virus-specific CD8 T cells with predominant 2B4^+^CD160^−^PD-1^+^ or 2B4^+^CD160^+^PD-1^+^ CD8 T-cell populations. These data suggest that cells expressing several co-inhibitory molecules are more profoundly impaired than cells expressing only one co-inhibitory molecule. Along the same line, PD-1 signaling blockade was on average more potent than CD160 signaling blockade in restoring virus-specific CD8 T-cell proliferation. Interestingly, the combined anti-CD160/anti-PD-L1/2 treatment showed additive effects only on virus-specific CD8 T cells containing both 2B4^+^CD160^−^PD-1^+^ or 2B4^+^CD160^+^PD-1^−^ CD8 T-cell populations, but not on virus-specific CD8 T cells co-expressing PD-1 and CD160. We postulate that cells expressing both co-inhibitory receptors might be intrinsically impaired, and their functions might be only partially reversible. Of note, PD-1 mean fluorescent intensity (MFI) was not significantly different between 2B4^−^CD160^−^PD-1^+^ and 2B4^+^CD160^+^PD-1^+^ CD8 T-cell populations (Fig. S5), suggesting that the difference in the restoration of proliferation capacity was not due to PD-1 expression level but likely on a different intrinsic capacity of the cells to proliferate.

Taken together, these data suggest that the functional restoration induced by the blockade of several co-inhibitory molecules may be incomplete and that cells expressing several co-inhibitory molecules are more profoundly impaired than cells expressing only one co-inhibitory molecule.

Consistently with a previous study [39], we showed that PD-1 expression was up-regulated following T-cell activation and proliferation. However, CD160 expression was down-regulated following T-cell activation and proliferation. Thus, PD-1/PD-1-Ligand blockade may have an effect on both cells expressing PD-1 at the time of stimulation and on proliferating cells while CD160 blockade may only act on cells expressing CD160 at the time of stimulation. The differences in CD160 and PD-1 expression may contribute to understand 1) the higher potency observed of the PD-1/PD-1-Ligand blockade as compared to CD160 blockade in restoring virus-specific CD8 T-cell proliferation and 2) the absence of correlation between the level of restoration of virus-specific CD8 T-cell proliferation capacity induced by PD-1/PD-L1 blockade and the *ex vivo* proportion of PD-1-expressing CD8 T cells.

Interestingly, the 2B4^−^CD160^−^PD-1^+^ CD8 T-cell population was significantly enriched in Flu-specific CD8 T cells as compared to EBV or CMV-specific CD8 T cells, and had higher IL-2 production capacity than all the other populations investigated. Furthermore, the 2B4^−^CD160^−^PD-1^+^ CD8 T-cell population expressed similar level of PD-1 (MFI) compared to the 2B4^+^CD160^+^PD-1^+^ CD8 T-cell population. The percentage of 2B4^−^CD160^−^PD-1^+^ CD8 T-cell population expressing EOMES, Tbet and CD57 was significantly lower than 2B4^+^CD160^−^PD-1^+^ and 2B4^+^CD160^+^PD-1^+^ CD8 T-cell populations. These data suggest that 2B4^−^CD160^−^PD-1^+^ CD8 T cells may be less functionally impaired than 2B4^+^CD160^−^PD-1^+^ and 2B4^+^CD160^+^PD-1^+^ CD8 T-cell populations.

In conclusion, while accumulation of different immune check point blockers is clearly associated with progressive dysfunction, the present study demonstrates that CD160-mediated regulation of CD8 T-cell functions is independent of PD-1 expression thus providing evidence that CD160 contributes to the regulation of CD8 T-cell functions.

## Materials and Methods

### Study group, ethics statement and cell isolation

Forty subjects were recruited in this study. Blood samples were obtained at the local blood bank (Centre de transfusion sanguine (CTS), Lausanne, Switzerland). The use of samples used in this study was approved by the Institutional Review Board of the CTS, and all subjects gave written informed consent. Only individuals with no sign of HIV, HAV, HBV and HCV infections were included. Blood mononuclear cells were isolated as previously described [40].

### Antibodies

The following monoclonal antibodies (mAbs) were used in different combinations. CD8-PB, CD3-APC-H7, PD-1-PECy7, PD-1-PB, IFN-γ-AF700, IL-2-PE, CD4-PB, granzyme B-AF700, perforin-APC were purchased from Becton Dickinson (BD, San Diego, CA), CD45RA-ECD, CD4-ECD from Beckman Coulter (Fullerton, CA, USA), CCR7-FITC from R&D Systems (Minneapolis, MN, USA), 2B4-PECY5.5, CD160-APC, CD160-PE from BioLegend (San Diego, CA, USA), CD4-eFluor650NC, CD8-eFluor625NC from eBioscience.

### *Ex vivo* analyses of CD8 T cells

Cryo-preserved blood mononuclear cells (1–2×10^6^) cells were washed, stained (30 min; 4°C) for dead cells using the Aqua LIVE/DEAD stain kit (Invitrogen) and, when required, stained with appropriately tittered peptide-MHC class I multimer complexes at 4°C for 30′ in Ca2^+^-free media as described [24]. Cells were then washed and stained (30 min; 4°C) with the following mAbs: CD3, CD8, CD4, PD-1, CD160 and 2B4.

### Virus-specific CD8 T-cell proliferation

Overnight-rested cryopreserved blood mononuclear cells (10^6^ in 1 ml of complete medium) were stained with 0.25 µM 5,6-carboxyfluorescein succinimidyl ester (CFSE, Molecular Probes, USA) as previously described [41], and stimulated with CMV, EBV or Flu peptides (1 µg/ml), 200 ng/ml of SEB (positive control; Sigma-Aldrich) or left unstimulated (negative control) in presence or in absence of anti-CD160 (2 µg/ml; MBL) and/or anti-PD-L1/2 (10 µg/mL) [13]. At day 6, cells were harvested and stained (4°C; 20 min) using the violet or aqua LIVE/DEAD stain kit (Invitrogen) and Abs (4°C; 30 min) to CD3, CD4, CD8. Frequencies of proliferating CFSE^low^ CD8 T cells were assessed by flow cytometry.

### Evaluation of the kinetic of CD160 and PD-1 expression on CD8 T cells after activation and expansion

Cryopreserved blood mononuclear cells (10^6^ in 1 ml of complete medium) were stained with 0.25 µM 5,6-carboxyfluorescein succinimidyl ester (CFSE, Molecular Probes, USA) as previously described [41], and stimulated with anti-CD3/anti-CD28 magnetic beads (Dynabeads, Invitrogen). The expression of CD160 and PD-1 on CD8 T cells was evaluated after 0, 24, 48, 72 and 120 hours of stimulation by anti-CD3/anti-CD28 magnetic beads. CD8 T-cell proliferation was also evaluated by CFSE dilution.

### Intracellular cytokine staining (ICS)

PBMC were stimulated overnight in complete media (RPMI (Invitrogen), 10% fetal calf serum (FCS; Invitrogen), 100 µg/ml penicillin, 100 unit/ml streptomycin (BioConcept)) with *Staphyloccocus enterotoxin B* (SEB; 250 ng/mL) or left unstimulated (negative control) in the presence of Golgiplug (1 µl/ml; BD) and anti-PD1, anti-CD160 and anti-2B4 mAbs. At the end of the stimulation period, cells were washed, stained (20 min; 4°C) for dead cells using the Aqua LIVE/DEAD stain kit (Invitrogen), permeabilized (20 min; 20°C) (Cytofix/Cytoperm, BD) and stained (30 min; 20°C) with mAbs to CD3, CD8, CD45RA, CCR7, IFN-γ and IL-2 [42].

### Perforin and granzyme B expression assessment

PBMC were washed, stained (20 min; 4°C) for dead cells using the Aqua LIVE/DEAD stain kit (Invitrogen). Cells were then washed and stained (30 min; 4°C) with the following mAbs: CD3, CD8, PD-1, 2B4, CD160, CCR7 and CD45RA. Cells were then permeabilized (20 min; 20°C) (Cytofix/Cytoperm, BD) and stained (30 min; 20°C) with mAbs to perforin and granzyme B [23].

### CD45RA, CCR7, EOMES, T-bet and CD57 expression assessment

PBMC were washed, stained (20 min; 4°C) for dead cells using the Aqua LIVE/DEAD stain kit (Invitrogen). Cells were then washed and stained (30 min; 4°C) with the following mAbs in different combinations: CD3, CD8, PD-1, 2B4, CD160 and CD57 or CD3, CD8, PD-1, 2B4, CD160, CCR7 and CD45RA. Cells were then permeabilized (1 h; 4°C) (Foxp3 Fixation/Permeabilization Kit; eBioscience) and stained (30 min; 4°C) with mAbs to EOMES and T-bet.

### Proliferation of CD8 T-cell populations

Cryopreserved CD8 T cells (30–40×10^6^ cells) previously selected by MACS cell separation (Miltenyi kit) were washed, stained (20 min; 4°C) for dead cells using the Aqua LIVE/DEAD stain kit (Invitrogen). Cells were then washed and stained (30 min; 4°C) with the following mAbs: CD8, PD-1, 2B4, CD160, CD45RA, CCR7. CD8 T-cell populations were sorted by BD FACSAria III on the basis of 2B4, PD-1 and CD160 expression after exclusion of naïve T cells (CD45RA^+^CCR7^+^). Sorted populations were labeled with CFSE and stimulated with viral peptides (1 µg/mL) in the context of irradiated (40 Gy) autologous CD8-depleted PBMCs (ratio CD8/feeder cells 1∶10) or in anti-CD3 (10 ug/ml) and anti-CD28 (0.5 ug/ml) mAbs coated plate or left unstimulated. Proliferation was assessed at day 6 by flow cytometry. Cells were washed, stained (20 min; 4°C) for dead cells using the Aqua LIVE/DEAD stain kit (Invitrogen). Cells were then washed and stained (30 min; 4°C) with anti-CD8 and anti-CD3 mAbs.

### Assessment of CD160 and CD160-TM expression

CD160 and CD160-TM expression were assessed both *ex vivo* and after *in vitro* expansion. To assess their expression *ex vivo* PBMC were washed, stained (20 min; 4°C) for dead cells using the Aqua LIVE/DEAD stain kit (Invitrogen). Cells were then washed and stained (30 min; 4°C) with the following mAbs: CD8, CD3, CD56, CD160, CD160-TM (rabbit unconjugated). Cells were then washed and stained (30 min; 4°C) with anti-rabbit secondary Ab. To assess their expression after *in vitro* expansion PBMC were labeled with CFSE and stimulated with IL-15 (50 ng/mL) or with anti-CD3 (10 ug/ml) and anti-CD28 (0.5 ug/ml) mAbs coated plate. CD160 and CD160-TM expression were assessed at day 6 by flow cytometry. Cells were washed, stained (20 min; 4°C) for dead cells using the Aqua LIVE/DEAD stain kit (Invitrogen). Cells were then washed and stained (30 min; 4°C) with the following mAbs: CD8, CD3, CD56, CD160, CD160-TM (rabbit unconjugated). Cells were then washed and stained (30 min; 4°C) with anti-rabbit secondary Ab.

### Flow cytometry analyses

Cells were fixed with CellFix (BD), acquired on an LSRII SORP (4 lasers: 405, 488, 532 and 633 nm) and analyzed using FlowJo (version 8.8.2) (Tree star Inc, Ashland, OR, USA) and SPICE 4.2.3. When required, analysis and presentation of distributions was performed using SPICE version 5.1, downloaded from <http://exon.niaid.nih.gov/spice> [43]. The number of lymphocyte-gated events ranged between 5×10^5^ and 10^6^ in the flow cytometry experiments.

### Statistical analyses

Statistical significance (*P* values) was obtained using one-way ANOVA (Kruskal-Wallis test) followed by Student's *t* test for multiple comparisons or a Spearman rank test for correlations using GraphPad Prism version 5.0 (San Diego, CA). Statistical analyses of global cytokine profiles (pie charts) were performed by partial permutation tests using the SPICE software as described [43].

## Supporting Information

Figure S1 **Expression levels of 2B4, CD160 and PD-1 on CD8 T cells and fluorescence minus one (FMO) for each of this molecule.** Flow cytometric profiles of CD8 T cells expressing PD-1, CD160 and 2B4 *ex vivo*. Representative example (healthy individual #304) of co-inhibitory molecules expression by CD8 T cells and the respective FMO for each molecule are shown.
(TIF)

Figure S2 **Mean fluorescent intensity (MFI) of CD2B4, CD160 and PD-1 on total CD8 T cells and virus-specific CD8 T cells.** The MFI of 2B4, PD-1 and CD160 expression on total CD8 T cells in 22 individuals was evaluated using polychromatic flow cytometry. **(A)** Cumulative data of the MFI of 2B4, PD-1 and CD160 expression within total CD8 T cells. **(B)** Ratio between the MFI of 2B4, PD-1 and CD160 expression in Flu, EBV and CMV-specific CD8 T cells and total CD8 T cells.
(TIF)

Figure S3 **CD160, but not PD-1 and/or 2B4 expression inversely correlates with CD8 T-cell proliferative capacity.** Correlations between proliferation index (CFSElow CD8 T-cell frequency/Multimer-specific CD8 T-cell frequency; x axes) and the virus-specific CD8 T-cell subsets distribution (percentage of Multimer-specific CD8 T cells expressing the 2B4, PD1 and CD160; y axes.
(TIF)

Figure S4 **Expression of EOMES, T-bet and CD57 on CD8 T-cell subsets discriminated by the expression of 2B4, CD160 and PD-1.** **(A)** Representative example (#1074) and cumulative analyses (n = 7) of EOMES and T-bet expression on distinct CD8 T-cell subsets. **(B)** Representative example (#1093) and cumulative analyses (n = 10) of CD57 expression on distinct CD8 T-cell subsets. Red bars correspond to mean ± SEM. Red stars indicate statistical significance (*P*<0.05). NS: not significant. Statistical significance (*P* values) was obtained using One-way ANOVA (Kruskal-Wallis test) followed by a paired Student's t-test.
(TIF)

Figure S5 **Mean fluorescence intensity (MFI) of 2B4, CD160 and PD-1 on CD8 T-cell subsets discriminated by the expression of 2B4, CD160 and PD-1.** Cumulative data of the mean fluorescence intensity of 2B4, CD160 and PD-1 on distinct CD8 T-cell subsets. Red bars correspond to mean ± SEM.
(TIF)

Figure S6 **Assessment of CD160 and CD160-TM expression.** The expression of CD160 and CD160-TM was evaluated by flow cytometry directly *ex vivo* or after *in vitro* expansion of NK cells (CD3^−^CD56^+^) and/or CD8 T cells (CD3^+^CD8^+^). **(A)** Representative flow cytometric profile of the CD160 and CD160-TM expression *ex vivo* by NK cells and CD8 T cells. **(B)** Representative flow cytometric profile of the CD160-TM expression after 5 days stimulation with IL-15 and/or anti-CD3/CD28 MAbs by NK cells and CD8 T cells.
(TIF)
