# Supplementary figures and images for: CD160-Associated CD8 T-Cell Functional Impairment Is Independent of PD-1 Expression
Source: PLoS Pathog. 2014 Sep 25;10(9):e1004380. doi: 10.1371/journal.ppat.1004380 (PMC4177992; doi:10.1371/journal.ppat.1004380)

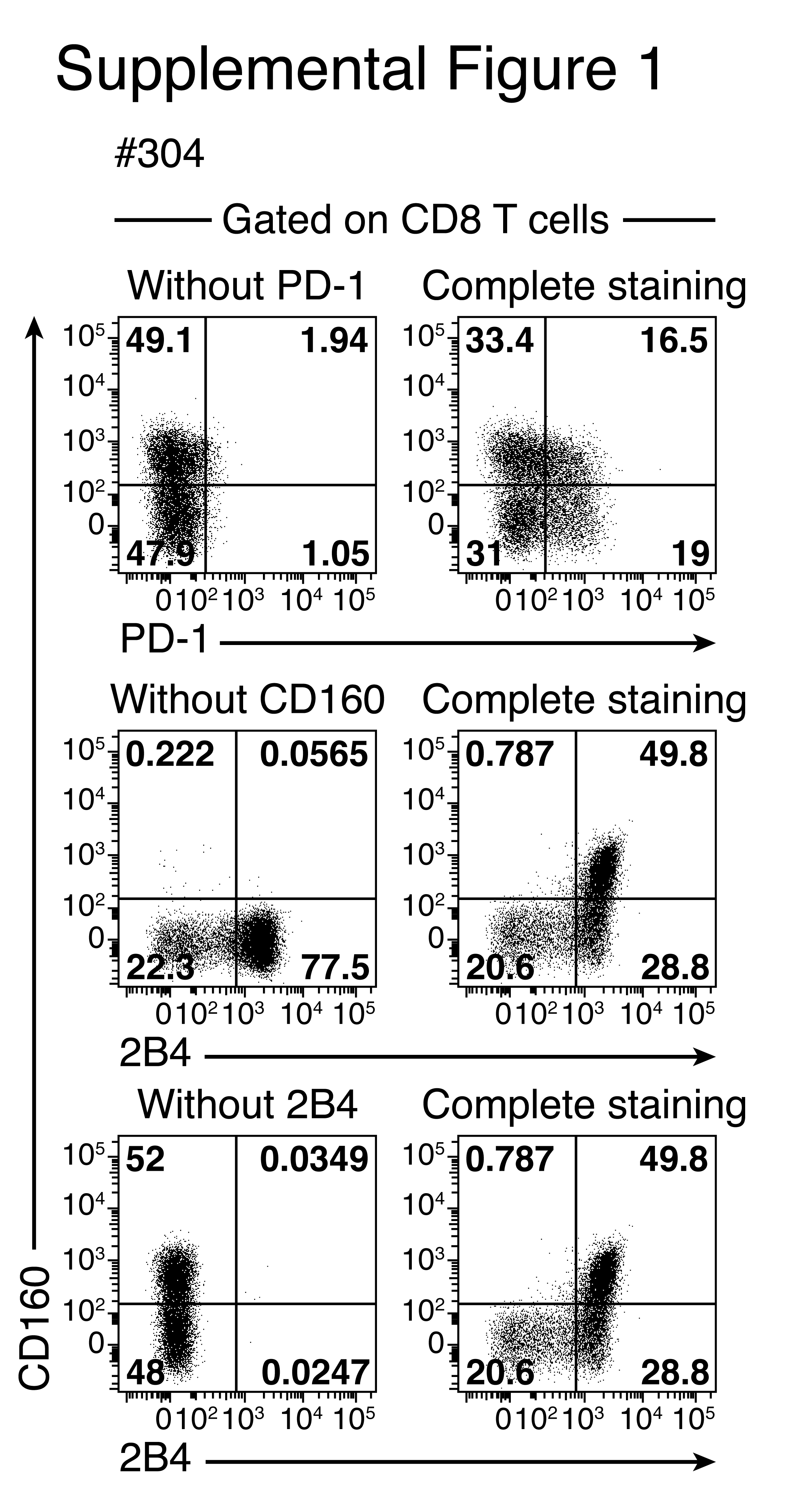

Supplement: Figure S1 — Expression levels of 2B4, CD160 and PD-1 on CD8 T cells and fluorescence minus one (FMO) for each of this molecule. Flow cytometric profiles of CD8 T cells expressing PD-1, CD160 and 2B4 ex vivo. Representative example (healthy individual #304) of co-inhibitory molecules expression by CD8 T cells and the respective FMO for each molecule are shown. (TIF) [file ppat.1004380.s001.tif]

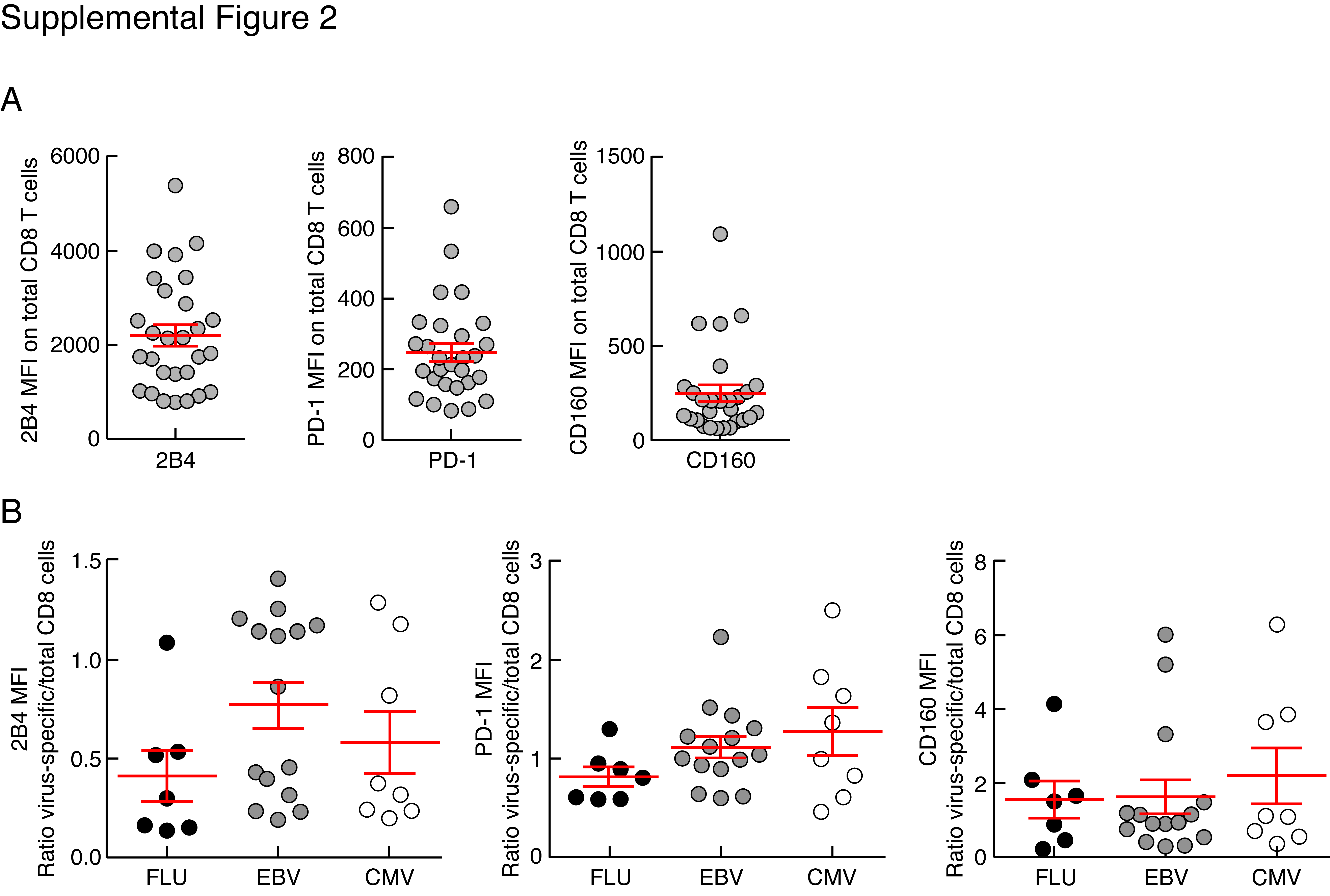

Supplement: Figure S2 — Mean fluorescent intensity (MFI) of CD2B4, CD160 and PD-1 on total CD8 T cells and virus-specific CD8 T cells. The MFI of 2B4, PD-1 and CD160 expression on total CD8 T cells in 22 individuals was evaluated using polychromatic flow cytometry. (A) Cumulative data of the MFI of 2B4, PD-1 and CD160 expression within total CD8 T cells. (B) Ratio between the MFI of 2B4, PD-1 and CD160 expression in Flu, EBV and CMV-specific CD8 T cells and total CD8 T cells. (TIF) [file ppat.1004380.s002.tif]

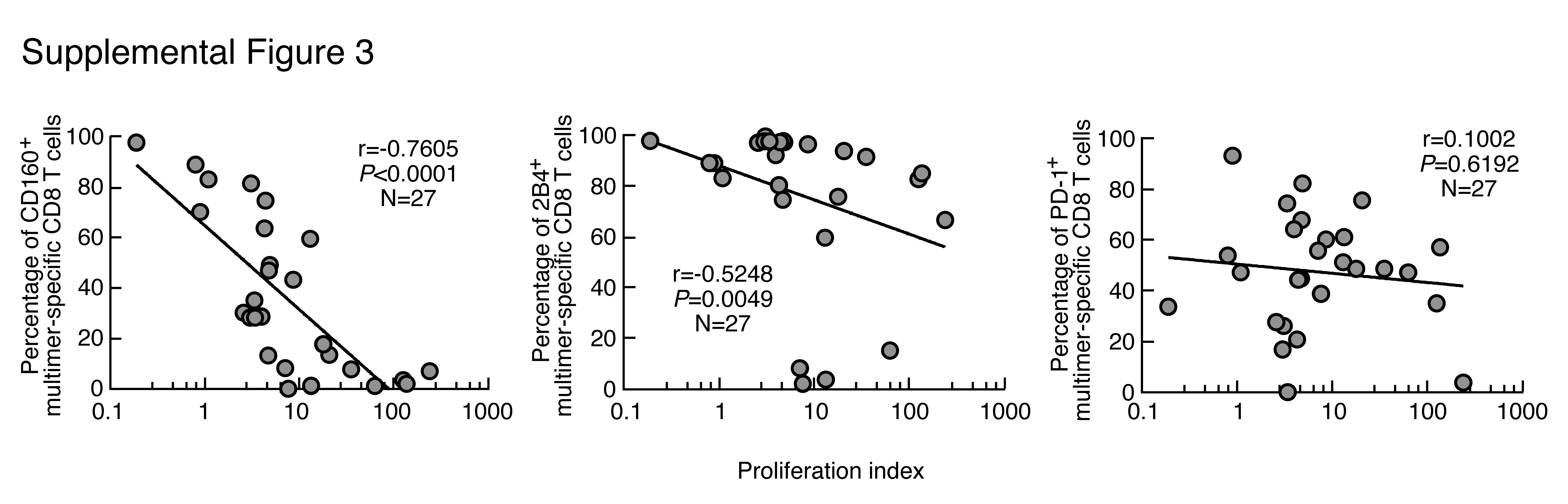

Supplement: Figure S3 — CD160, but not PD-1 and/or 2B4 expression inversely correlates with CD8 T-cell proliferative capacity. Correlations between proliferation index (CFSElow CD8 T-cell frequency/Multimer-specific CD8 T-cell frequency; x axes) and the virus-specific CD8 T-cell subsets distribution (percentage of Multimer-specific CD8 T cells expressing the 2B4, PD1 and CD160; y axes. (TIF) [file ppat.1004380.s003.tif]

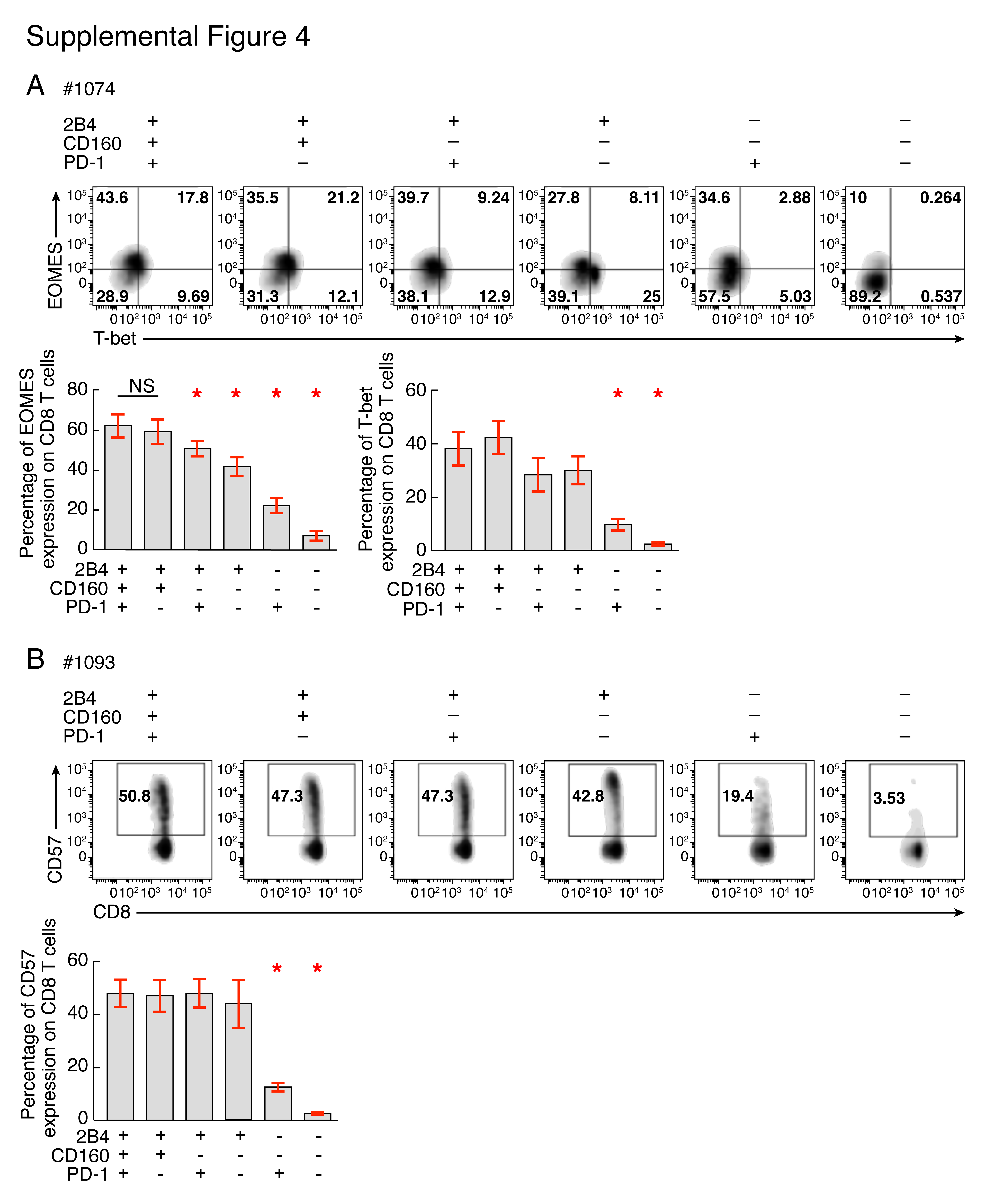

Supplement: Figure S4 — Expression of EOMES, T-bet and CD57 on CD8 T-cell subsets discriminated by the expression of 2B4, CD160 and PD-1. (A) Representative example (#1074) and cumulative analyses (n = 7) of EOMES and T-bet expression on distinct CD8 T-cell subsets. (B) Representative example (#1093) and cumulative analyses (n = 10) of CD57 expression on distinct CD8 T-cell subsets. Red bars correspond to mean ± SEM. Red stars indicate statistical significance (P<0.05). NS: not significant. Statistical significance (P values) was obtained using One-way ANOVA (Kruskal-Wallis test) followed by a paired Student's t-test. (TIF) [file ppat.1004380.s004.tif]

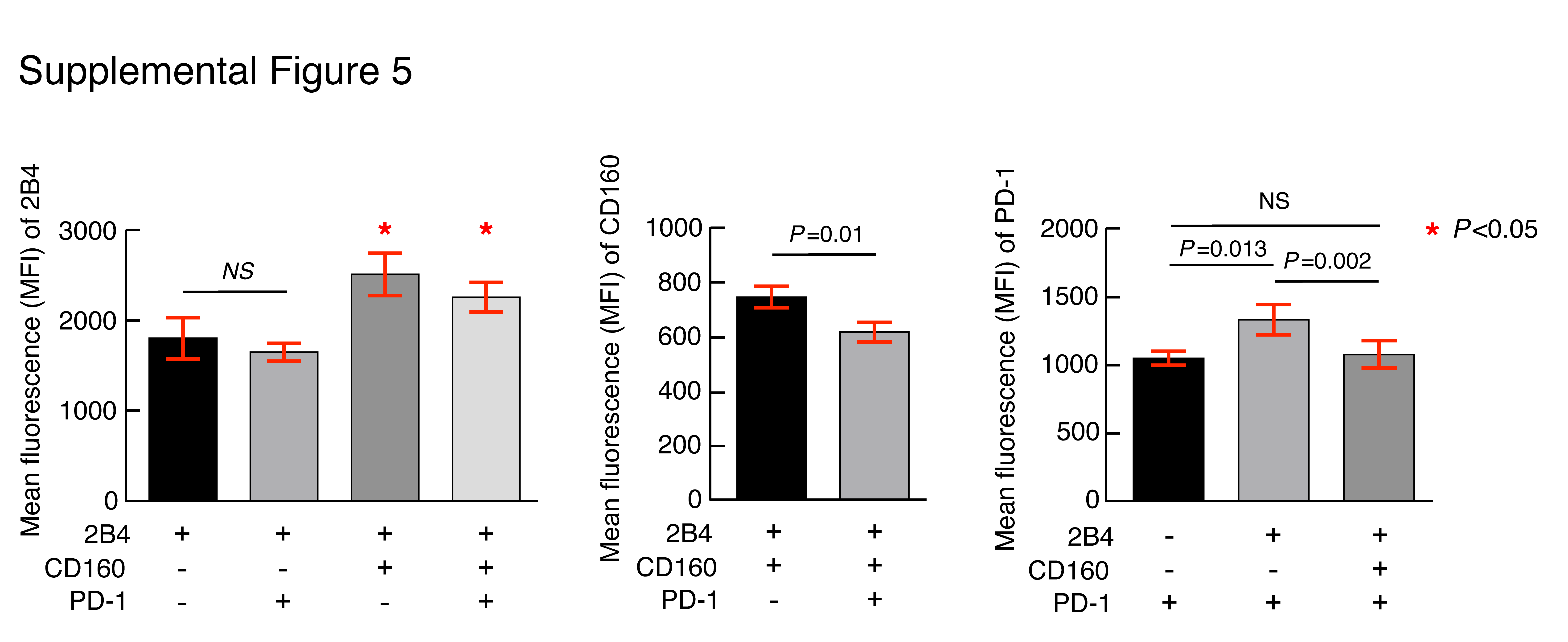

Supplement: Figure S5 — Mean fluorescence intensity (MFI) of 2B4, CD160 and PD-1 on CD8 T-cell subsets discriminated by the expression of 2B4, CD160 and PD-1. Cumulative data of the mean fluorescence intensity of 2B4, CD160 and PD-1 on distinct CD8 T-cell subsets. Red bars correspond to mean ± SEM. (TIF) [file ppat.1004380.s005.tif]

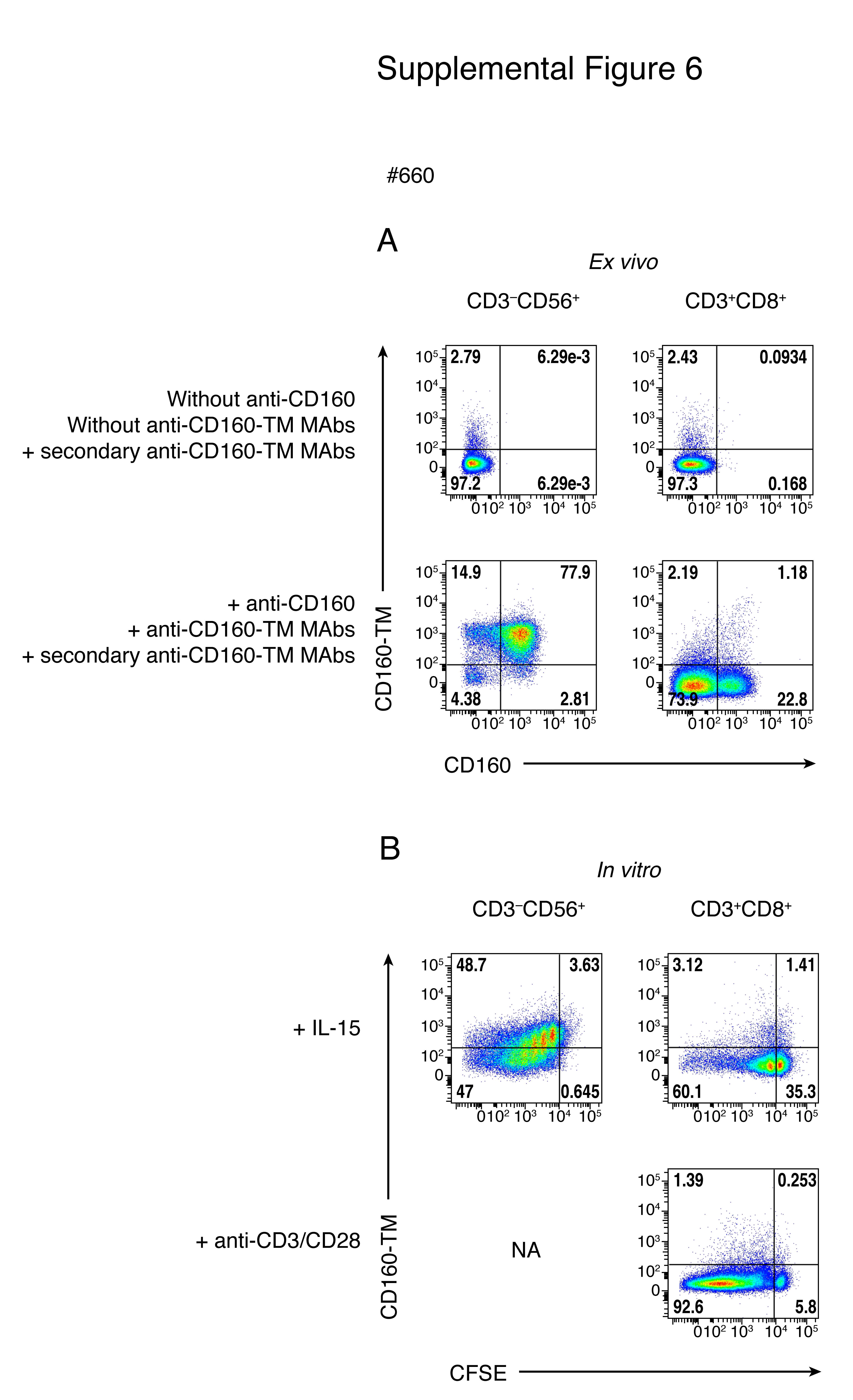

Supplement: Figure S6 — Assessment of CD160 and CD160-TM expression. The expression of CD160 and CD160-TM was evaluated by flow cytometry directly ex vivo or after in vitro expansion of NK cells (CD3−CD56+) and/or CD8 T cells (CD3+CD8+). (A) Representative flow cytometric profile of the CD160 and CD160-TM expression ex vivo by NK cells and CD8 T cells. (B) Representative flow cytometric profile of the CD160-TM expression after 5 days stimulation with IL-15 and/or anti-CD3/CD28 MAbs by NK cells and CD8 T cells. (TIF) [file ppat.1004380.s006.tif]
